# Oral health status of NCC cadets in Uttar Pradesh: A cross-sectional study

**DOI:** 10.6026/973206300221000

**Published:** 2026-02-28

**Authors:** Varsha Sharma, Priti Shukla, Shivesh Acharya, Kanika Singh Dhull, Mukul Sharma

**Affiliations:** 1 Department of Dentistry, Maharshi Vashishtha Autonomous State Medical College, Basti, Uttar Pradesh, India; 2 Department of Dentistry All India Institute of Medical Sciences, Raebareli, Uttar Pradesh, India; 3 Department of Pediatric and Preventive Dentistry, Kalinga Institute of Dental Sciences, KIIT University, Patia, Bhubaneswar - 751024, Odisha, India; 4 Department of Forensic Medicine and Toxicology, All India Institute of Medical Sciences, Raebareli, Uttar Pradesh, India

**Keywords:** Dental caries, debris index, National cadet corps (NCC), Oral Hygiene Index-Simplified (OHI-S), Decayed Missing Filled Teeth (DMFT)

## Abstract

National Cadet Corps (NCC) helps in nation building. There is paucity of information on their oral health status. So, a cross- sectional study was conducted on 165 NCC cadets, aged 13-18 years to assess their oral health, hygiene status and prevalence of dental conditions. Study population was divided in two groups based on age. Oral health screening was done using the Oral Hygiene Index- Simplified (OHI-S) and Decayed Missing Filled Teeth (DMFT) index. The Debris Index was found to be significantly higher. Mean DMFT among age group 13-15 was 0.3 ± 0.8 and among age group 16-18 was 0.68 ± 1.1. Overall, the oral hygiene practice of the cadets was found to be poor.

## Background:

National cadet corps (NCC) is a renowned wing of Indian armed forces (tri-service organization which includes army, navy and air- force) which is voluntary for students at schools and colleges [1]. NCC aims towards youth development by grooming the young minds and developing leadership, character, discipline and patriotism among them. Students learn time management skills due to adherence to a strict schedule are also trained in handling small arms, map reading and adventurous activities like mountaineering, trekking and sailing [2]. The oral health of NCC cadets is influenced by dietary habits, personal practices and stress factors. A combination of cadets' occupied training commitments and limited knowledge of oral hygiene contributes to inadequate oral care and the subsequent development of oral diseases. Recognizing that oral health is a vital part of overall well-being, its significance is particularly pronounced among the youth of our nation [3]. Therefore, it is of interest to illuminate the oral health status and disparities among NCC cadets, delving into the challenges they face and the potential impacts on their overall health.

## Materials and Methodology:

## Study design and population:

This cross-sectional epidemiological study was conducted as a dental screening camp for NCC Cadets. The aim was to investigate the oral health status of NCC cadets aged between 13-18 years. Ethical clearance was obtained from institutional committee.

## Sampling method and sample size:

Universal sampling was used. 165 NCC cadets who volunteered to participate were included in the study and divided into two groups according to age, Group 1 - Younger Cadets (13-15 years) and Group 2 - Older cadets (16-18 years). Written parental consent was collected prior to examination through the camp administrator.

## Data collection:

To evaluate the oral health status of the cadets, both the Oral Hygiene Index-Simplified (OHI-S) and Decayed Missing Filled Teeth (DMFT) index were used. All examinations were conducted by a single examiner, with assistance from a trained recording dentist. The collected data was coded and then entered into an Excel sheet.

## Statistical analysis:

The data was analysed using SPSS Statistical software version 27.0. Chi-square tests and analysis of variance (ANOVA) were applied to the relevant variables. A 95% confidence interval was used and the significance level was set at 5%.

## Results:

The socio-demographic distribution of 165 cadets has been documented (Table 1). The participated cadets belonged to age group between 13 to 18 years and majority were males (93%). The mean age was 16.5 years among the two groups. The oral health status through Decayed, Missing and Filled Teeth (DMFT) showed 32% of cadets with DMFT>0 and OHIS showed poor oral hygiene practise among 42% cadets (Table 1). Prevalence of caries was non-significant among the two age groups (P = 0.215) (Table 2). No significant difference between the two genders was found (P = 0.556). Table 3 suggests significantly higher value of Debris Index in older cadets as compare to younger cadets (P = 0.009). Whereas, Calculus Index and OHI found to be non-significant in both the groups (P = 0.848, P = 0.066 respectively). The prevalence of various oral health conditions as recorded was tabulated (Table 4).

## Discussion:

National Cadets Corps (NCC) grooms the youth - 'The Leaders of Tomorrow' - into disciplined and patriotic citizens. NCC aims at developing character, comradeship, discipline and a secular outlook, the spirit of adventure and ideals of selfless service amongst young citizens. NCC cadets shoulder extra responsibilities by taking active participation in social awareness initiatives, addressing environmental issues, providing disaster relief, engaging in adventure and sports activities and contributing to various nation-building efforts [1]. Thus it becomes the duty of clinicians, to create awareness among them towards preventive measures about their oral health and hygiene [4]. During the study, it was observed that the daily tooth brushing practices were neglected among most of them due to early hours of parades, drills and exercises. A thick layer of plaque deposits was present in them regardless of their age. A significant high level of Debris Index (P = 0.009) was found among older cadets ranging between 16-18 years [5]. 32% of cadets had DMFT score>0 similar to that observed by Broadbent and Thompson [6]. According to World Health Organisation, children between age group 10-19 undergo some developmental changes which might affect their behaviour [7]. It is quite possible that the younger children comply better with the instructions as compared to older one [8]. A similar result was seen in our study as Group 1 showed willingness to follow daily oral hygiene practices in the camp as compared to older cadets of Group 2. Older age group children easily detach from the instructions from the authorities thereby creating a need for constant motivation for daily oral hygiene practices [9]. Dental caries followed by periodontitis then malocclusion were the most prevalent oral diseases recorded. Alarmingly, 4% cadets reported with grade I Oral Submucous Fibrosis (OSMF) and gave a history of chewing sweet supari. OSMF is a premalignant condition associated with chewing of areca nut [10, 11]. Children develop the habit of chewing these psycho-stimulating products as they are easily accessible in various multi-coloured attractive pouches in the market [12]. Other contributing factors include levels of awareness, household environment, peer pressure and low cost availability of such substances [13]. A similar number (4%) of cadets presented their concern of the poor aesthetics which was due to fluorosis. Fluorosis is generally caused by excessive fluoride intake during critical period of development of tooth [14]. Increasing the awareness regarding different treatment modalities available for fluorosis can prevent a decrease in self-esteem [15]. Children should also be educated regarding the higher risk of caries in severe form of fluorosis [16]. 2% cadets reported with painful aphthous ulcers. This could be the result of severe stress they face at the camp as NCC combined annual training camp (CATC) is meticulously scheduled for 9-10 days. 3% cadets reported with poor hygiene related to pericoronitis. This condition was more prevalent among individuals with erupting third molars during these age groups and requires timely intervention [17].

## Conclusion:

We show that NCC cadets had very poor knowledge about oral hygiene and those who knew were reluctant to practice it. Hence in a facility, apart from sculpting the adolescents mentally and physically, their oral health and overall health also needs to be taken care of. NCC authorities should take the initiative to promote oral health programs among the cadets by providing timely oral health check- ups and educative programmes.

## Figures and Tables

**Table 1:** Distribution of Age, Gender and OHI-S Scores.

**Variables**		**Frequency (n)**	**Percentage (%)**
Age group	13-15	41	25%
	16-18	124	75%
Gender	Male	153	93%
	Female	12	7%
DMFT	0	113	68%
	>0	52	32%
OHI score	Good (0-1.2)	11	7%
	Fair (1.3-3.0)	85	51%
	Poor (3.1-6.0)	69	42%

**Table 2:** Comparison of two groups with decayed teeth, missing teeth, decayed, missing, filled teeth.

**Age Group**	**DT**			**MT**			**FT**				**DMFT**	
	Mean ± SD	Lower limit	Upper limit	Mean ± SD	Lower limit	Upper limit	Mean ± SD	Lower limit	Upper limit	Mean±SD	Lower limit	Upper limit
13-15	0.34 ± 0.7	0	3	0	0	0	0	0	0	0.34 ± 0.77	0	3
16-18	0.61 ± 1.1	0	5	0.07 ± 0.37	0	3	0.02 ± 0.18	0	2	0.68 ± 1.14	0	5
SD: Standard Deviation,
DT: Decayed Teeth,
FT: Filled Teeth,
DMFT: Decayed, Missing, Filled Teeth

**Table 3:** Comparison of two groups with Debris Index, Calculus Index, Oral Hygiene Index Simplified (OHI-S)

	**DI-S**			**CI-S**			**OHI-S**		
Age Group	Mean + SD	Lower limit	Upper limit	Mean + SD	Lower limit	Upper limit	Mean + SD	Lower limit	Upper limit
13-15	2.42 ± 0.6	1	3	0.463 ± 0.6	0	2	2.87 ± 1.2	0	5
16-18	2.57 ± 0.5	2	3	0.565 ± 0.7	0	3	3.14 ± 1.1	2	6
Chi square value	9.48			0.805			10.35		
p value	0.009			0.848			0.066		
SD: Standard deviation,
* highly significant,
DI-S: Debris Index Simplified,
CI-S: Calculus Index Simplified,
OHI-S: Oral Hygiene Index Simplified.

**Table 4:** Prevalence of oral diseases

**Dental Caries**	**29.60%**
Periodontal Disease	24.20%
Malocclusion	15.40%
Oral Submucous Fibrosis (OSMF)	4.20%
Fluorosis	4.20%
Pericoronitis	3%
Aphthous Ulcer	1.80%
Retained Deciduous	1.80%
Geographic Tongue	1.20%
Ellis Class II Fracture	1.20%
